# Regulation of Synaptic Transmission at the *Caenorhabditis elegans* M4 Neuromuscular Junction by an Antagonistic Relationship Between Two Calcium Channels

**DOI:** 10.1534/g3.114.014308

**Published:** 2014-11-04

**Authors:** Mark Steciuk, Mi Cheong Cheong, Christopher Waite, Young-Jai You, Leon Avery

**Affiliations:** * Department of Molecular Biology, University of Texas Southwestern Medical Center, Dallas, Texas 75390-9148; † Department of Physiology and Biophysics, Virginia Commonwealth University, Richmond, Virginia 23298-0551; ‡ Department of Biochemistry and Molecular Biology, Virginia Commonwealth University, Richmond, Virginia 23298-0614

**Keywords:** behavior, feeding, synaptic transmission, calcium channels, BK channel

## Abstract

In wild-type *Caenorhabditis elegans*, the synapse from motor neuron M4 to pharyngeal terminal bulb (TB) muscles is silent, and the muscles are instead excited by gap junction connections from adjacent muscles. An *eat-5* innexin mutant lacking this electrical connection has few TB contractions and is unable to grow well on certain foods. We showed previously that this defect can be overcome by activation of the M4 → TB synapse. To identify genes that negatively regulate synaptic transmission, we isolated new suppressors of *eat-5*. To our surprise, these suppressors included null mutations in NPQR-type calcium channel subunit genes *unc-2* and *unc-36*. Our results are consistent with the hypothesis that Ca^2+^ entry through the NPQR-type channel inhibits synaptic transmission by activating the calcium-activated K^+^ channel SLO-1, thus antagonizing the EGL-19 L-type calcium channel.

*Caenorhabditis elegans* has been a powerful engine for the discovery of molecules involved in synaptic transmission (Richmond 2005). This is because, in addition to capabilities it shares with some other model organisms, it has two unique advantages. First, in the laboratory worms barely need their nervous systems, so that mutants with profoundly depressed synaptic transmission are viable and fertile (Richmond 2005). Second, there is a powerful selection for such mutants: survival in the presence of acetylcholinesterase inhibitors such as lannate (Brenner 1974) or aldicarb (Rand 2007). This allows the easy identification of genes that are necessary for normal levels of synaptic transmission. Unfortunately, there has not been a comparably simple method for identifying genes whose products inhibit synaptic transmission.

Some years ago we described phenomena that might lead to such a method. The action of the *C. elegans* feeding organ, the pharynx, depends on contraction of groups of muscles in the anterior, the corpus, and the posterior, the terminal bulb (TB) (Avery and You 2012). These muscles are electrically coupled, and TB muscles are normally excited by excitation spreading from the corpus (Starich *et al.* 1996). In mutants that lack the innexin EAT-5, this coupling is lost. Consequently, the TB contracts less frequently than in the wild-type (Chiang *et al.* 2006). These *eat-5* mutants are almost unable to grow on the *Escherichia coli* strain DA837 but grow well on *E. coli* HB101 (Avery and Shtonda 2003; Chiang *et al.* 2006).

In many nematode species, TB muscles are excited by the M4 motor neuron. In *C. elegans* this synapse is present, as revealed by synaptobrevin::GFP (green fluorescent protein) fusions, but it is electrophysiologically undetectable and functionally silent (Chiang *et al.* 2006). The BK calcium-activated potassium channel SLO-1 inhibits synaptic transmission in *C. elegans* (Wang *et al.* 2001). We found that in a mutant that lacks SLO-1, the M4 → TB neuromuscular junction is electrophysiologically active and functional. The rate of *eat-5; slo-1* TB contraction, although not restored to the wild type, is about double that of an *eat-5* single mutant (Chiang *et al.* 2006). We show here that this is sufficient to allow growth on *E. coli* DA837.

This finding suggested an efficient method of isolating mutants that increase synaptic transmission. Because they grow well on HB101, *eat-5* worms can easily be obtained in large numbers, mutagenized, then their progeny tested for growth on DA837. Using this method, we screened 27,000 mutagenized haploid genomes and isolated 43 suppressors of *eat-5* (abbreviated *sef*, for Suppressor of Eat-Five), which define about a dozen complementation groups. These include *unc-2* and *unc-36*, which encode subunits of one of the three *C. elegans* voltage-gated calcium channels. The α_1_ subunit, UNC-2, is most similar to N, P/Q, and R-type mammalian channels. This surprised us, because UNC-2 has previously been shown to act positively in synaptic transmission at the body muscle neuromuscular junction (Richmond *et al.* 2001), where it is thought to be the main source of Ca^2+^ to trigger vesicle fusion (Richmond 2005). We show here that the negative action of UNC-2/UNC-36 at the M4 neuromuscular junction requires SLO-1 and that the increased TB pumping seen in *unc-2* mutants is blocked by a mutation in the L-type voltage-gated calcium channel EGL-19. The observations are consistent with a model in which the NPQR-type and L-type channels play antagonistic roles in transmission, the L-type channel serving as the main source of Ca^2+^ to stimulate vesicle fusion, and the NPQR-type channel acting via the BK channel to truncate the depolarization necessary for L-type channel activation.

## Materials and Methods

### Strains

Worms were maintained on *E. coli* growing on NGMSR medium (Davis *et al.* 1995). We used two *E. coli* strains. DA837 (Davis *et al.* 1995) is the restrictive strain for *eat-5* growth, and HB101 (Boyer and Roulland-Dussoix 1969) the permissive (Avery and Shtonda 2003). The *eat-5* allele used throughout this work is *ad1402*, a small deletion (Chiang *et al.* 2006). *eat-5* worms were maintained routinely on HB101. For other genes, the following mutations were used, and are designated in the text with just the gene name: *cfi-1(ky651)*, *eat-18(ad1110)*, *eat-2(ad465)*, *egl-19(n582)*, *unc-36(e251)*, *slo-1(js379)*, *unc-2(e55)*, and *cca-1(ad1650)*. We also used *unc-2(mu74)* and *slo-1(ad1614)* for some experiments, and the allele name is given explicitly in these cases.

### Video microscopy

L1 worms were collected between 1 and 3 hr after hatching, mounted with DA837 bacteria on agar pads, and observed on a Zeiss Axio Imager A2 microscope through a 63× NA 1.4 PlanApo objective with DIC optics. Recordings were made with a Point Gray Flea3 1.3MP Mono USB 3.0 camera. Supporting Information, File S1, File S2, and File S3 was downsampled to 640 × 480 with Apple iMovie.

### Selection for growth

Synchronized *eat-5* L4 hermaphrodites were mutagenized with 50 mM ethyl methanesulfonate (EMS) in M9 buffer for 4 hr (Sulston and Hodgkin 1988), then allowed to grow to adulthood on HB101. F1 eggs were prepared by basic hypochlorite treatment (Emmons *et al.* 1979) of the gravid P0 adults and grown to adulthood on HB101, and then F2 eggs were similarly prepared and placed on DA837 plates. The number of viable F2s was measured by placing an aliquot of the egg suspension on HB101.

### Screen for weak suppressors

F1 progeny of mutagenized *eat-5* P0s were prepared as described previously. A single gravid adult was placed on each of 5128 DA837-seeded plates (10,256 mutagenized genomes), then removed after laying eggs for 1 d. F2 worms that reached the L4 or adult stage by the fifth day after plating of the F1 were picked to a new DA837 plate. Lines that consistently threw large numbers of worms that grew to the L4 stage by the fifth day over the course of three generations were called suppressors, after which they were transferred to HB101 plates for further analysis. Only one line per F1 was kept. Ultimately, we isolated 43 such suppressed strains. Eighteen of these were so weak as to be impractical to work with, but we were ultimately able to analyze 25 at least partially. In summary, between the selection and the screen, we isolated a total of 56 mutations that allowed improved growth of *eat-5* on DA837 and further analyzed 38.

### Backcrossing

We typically backcrossed *sef* to *eat-5* as follows. First, *eat-5; sef* hermaphrodites were crossed with *eat-5* males on HB101. Their *eat-5; sef/+* male progeny were then crossed with *eat-5* hermaphrodites on HB101 under conditions that result in close to 100% outcrossing and several (typically 8) L4 hermaphrodite progeny were picked from this cross to DA837, one to a plate. Half of the progeny of this cross are expected to be *eat-5; sup/+*. (Two suppressors, both alleles of *unc-2*, were X-linked, so that the progeny of the first backcross were *eat-5; unc-2/O* and all rather than half of the progeny of the second cross *eat-5; unc-2/+*.) *eat-5; sef/+* worms are recognized by the production of progeny that escape L1 arrest, some or all of which will be homozygous for the suppressor, depending on whether it is dominant or recessive. This scheme includes two backcrosses. It dilutes unlinked autosomal mutations fourfold and X-linked mutations twofold. It was repeated up to three times for a total of up to six backcrosses.

### Dominance and complementation tests and genetic mapping

To test for dominance *eat-5; sef* or *eat-5; sef/+* males were mated with *dpy-5eat-5* hermaphrodites on DA837. If more cross-progeny hermaphrodites escaped arrest than on a concurrent *eat-5* × *dpy-5eat-5* control cross, we deduced dominance. For the complementation test between two recessive suppressors *sefA* and *sefB*, we mated *eat-5; sefA/+* males with *dpy-5eat-5; sefB* or *eat-5unc-13; sefB* on DA837; failure of complementation was deduced if more cross-progeny hermaphrodites escaped arrest than on a concurrent *eat-5* × *dpy-5eat-5; sefB* or *eat-5unc-13; sefB* control cross.

Most suppressor mutations have not been mapped genetically. Some mutations were found to be on *I* in the course of constructing *dpy-5eat-5 sef* or *eat-5unc-13 sef* triple mutants, and as noted previously *unc-2* alleles were found to be X-linked on backcrossing. *unc-36 III* and *eat-2 II* alleles were recognized by complementation tests with existing mutations of genes that produce similar phenotypes. Finally, the dominant mutation *dod-6(ad1609)* was mapped to *III* as follows. *eat-5; ad1609* males were mated with the multiply marked strain DA438 (*bli-4 I; rol-6 II; daf-2vab-7 III; unc-31 IV; dpy-11 V; lon-2 X*) (Avery 1993), then the resulting males (*bli-4/eat-5 I; rol-6/+ II; daf-2vab-7/ad1609 III; unc-31/+ IV; dpy-11/+ V; lon-2/O X*) were mated with *eat-5* hermaphrodites under conditions that promote near-complete outcrossing. A total of 92 progeny of this cross were placed on individual DA837 plates and their self-progeny examined. We found that 51 of 92 threw Bli progeny; because *bli-4* is linked to *eat-5*, these were likely to be *eat-5* heterozygotes and were not further examined. A total of 25 of 92 produced progeny that arrested on DA837. The remaining 16 produced suppressed progeny and therefore must have received *ad1609* from their fathers. Of these, 9 threw Rol progeny, 0 threw Vab, 11 threw Unc, and 9 threw Dpy, showing *ad1609* to be autosomal, not tightly linked to *bli-4*, *rol-6*, *unc-31*, or *dpy-11*, and on *III* less than 20 centimorgans from *vab-7*.

### Genome sequencing and gene identification

The genomes of nine *eat-5; sef* suppressor strains isolated in the selection were sequenced, along with the parental *eat-5* single mutant strain, via Illumina sequencing. Sequences were aligned to the WS220 reference *C. elegans* genome with bowtie2 (Langmead and Salzberg 2012), variants called with the samtools/bcftools suite (Li *et al.* 2009), and effects on gene function predicted and variants filtered with snpEff and SnpSift (Cingolani *et al.* 2012). Further specific analyses used vcftools (Danecek *et al.* 2011), bedtools (Quinlan and Hall 2010), and custom scripts. These included scripts to look for small deletions, but aside from *eat-5(ad1402)* we found none in these mutants. Results were viewed with IGV (Robinson *et al.* 2011) and Microsoft excel.

We found in the nine sequenced mutants five *cfi-1* alleles, three *dod-6* alleles, and one allele of *slo-1*. Based on closely linked EMS-induced (*i.e.*, GC→AT) mutations, the five *cfi-1* alleles comprise four independent events (*i.e.*, one of the mutations was isolated twice) and the three *dod-6* alleles two independent events.

We also identified some of the mutations isolated in the screen by genome sequencing. In this case, we used the strategy of Zuryn *et al.* (2010). Ten mutations were backcrossed six times to the parental strain DA1402 as described previously, then the genomes of the backcrossed strains were sequenced and searched for clusters of potential EMS-induced (*i.e.*, G→A or C→T) mutations not present in DA1402. In this way we identified mutations in *cfi-1*, *eat-2*, *eat-18*, and *slo-1*, and obtained a list of candidates for some others.

An existing *cfi-1* allele, *ky651* (Shaham and Bargmann 2002), was shown to suppress *eat-5* by constructing *dpy-5eat-5cfi-1* and showing that it grows on DA837 and frequently has synchronized pharyngeal pumping. *cfi-1* mutations identified as *eat-5* suppressors failed to complement *cfi-1(ky651)* for this phenotype. Alleles of *unc-2*, *unc-36*, *eat-2*, and *slo-1* were identified by complementation tests with existing mutations and scored by the visible locomotion and feeding phenotypes of these mutations. *unc-2*, *unc-36*, *eat-2*, *eat-18*, and *slo-1* were confirmed as *eat-5* suppressors by construction of *eat-5* doubles with previously reported loss-of-function alleles *e55*, *mu74* (for *unc-2*), *e251* (*unc-36*), *ad465* (*eat-2*), *ad1110* (*eat-18*), and *js379* (*slo-1*). This test was not available for *dod-6*, since no mutant alleles have been reported previously, and the mutation we found is a likely gain-of-function. In this case, gene identification rests on our finding a *dod-6* mutation in two independently isolated suppressors with identical phenotypes, and on the genetic map location described previously.

### Estimation of mutation frequencies

If suppressor mutations arise in gene *X* at frequency *f* per EMS-mutagenized genome, then the frequency of *X*-bearing suppressors in the F1 is 2*f*. The frequency of suppressed F2s is *f*/2 for a recessive suppressor. We can thus estimate *f* as

$$\hat{f}=\frac{n}{2F1+F2/2}$$ (1)

Here F1 is the number of F1s in the weak suppressor screen, F2 is the number of F2s in the selection, and *n* is the total number of suppressors in gene *X* isolated in both the F2 selection and the F1 screen.

A better estimate is available when the number of *independent* suppressor gene *X* mutations is known, which was the case for *dod-6*, since we sequenced all alleles isolated. Then

$$\hat{f}=\frac{n_{i}}{G}$$ (2)

*n_i_* is the number of independent gene *X* suppressors, and *G* the effective number of genomes screened. *G* for the selection is calculated as shown in Table S1; for the screen, it is 2F1.

### Measurement of TB pump rate

TB pumps were measured using L1s that were between 30 min and 75 min from hatching, after eggs were collected as described (Emmons *et al.* 1979). TB pumps were counted using a 20× objective on a Zeiss microscope.

### Growth rate measurement

Five *C. elegans* L4 hermaphrodites from each strain were picked and transferred to a DA837-seeded plate to roughly match the developmental age. Next day, the five worms were moved individually to a new DA837-seeded plate. Plates were observed once a day until all food had been consumed.

### Generating transgenic lines

Fusion constructs were made using a two-step process adapted from previously described protocols (Horton *et al.* 1989; Hobert 2003). All polymerase chain reactions were performed using the Extend Long Template PCR Kit (Roche). DNA transformation was performed as described previously (Mello and Fire 1995). For all injections, a transcriptional fusion of a given promoter sequence (*snb-1*: pan-neuronal; *ceh-28*: M4, M2, extrapharyngeal cells; *myo-2*: pharyngeal muscle; *unc-4*: I5, extrapharyngeal cells; *egl-17*: M4, extrapharyngeal cells; *nlp-13*: M2, I2, NSM, M1, extrapharyngeal cells) fused to GFP was coinjected with the same promoter sequence fused to *unc-36* genomic DNA. An intestine-specific GFP marker (*odc-1* promoter transcriptionally fused to GFP, gift from Alan Chiang) was coinjected with promoters of *egl-17* and *ceh-28*. After injection, transgenic lines were isolated based on the GFP expression using an Olympus SZX12 GFP dissecting microscope. The GFP expression was further confirmed using Zeiss microscope with a 63× objective.

### Measurement of escape from arrest in transgenic strains

Because it is difficult to get a pure population of transgenic worms, we measured the effect of transgenes by estimating the number of worms that could escape arrest and reach L4 stage within 5 days. Transgenic L4 hermaphrodites were transferred individually to either DA837 or HB101 plates. Five days later, the percentage of transgenic progeny that reached the L4 stage or greater was recorded. To estimate the rate of escape from arrest, we also needed to know the transmission rate of the transgene (*i.e.*, the proportion of progeny of a transgenic worm that are themselves transgenic). We measured this by counting transgenic and non-transgenic worms that reached L4 stage within three days of placing a single L4 mother on an HB101 plate. Finally, we estimated relative escape from arrest using

$$\begin{array}{c} r=\frac{\left(1-f\right)t}{\left(1-t\right)f}\\ \mathrm{SE}M_{r}=r\sqrt{\frac{1}{N_{D}f\left(1-f\right)}+\frac{1}{N_{H}t\left(1-t\right)}} \end{array}$$ (3)

where

$$\begin{array}{c} t=\mathrm{transmission}\;\mathrm{rate}\;\mathrm{estimated}\;\mathrm{from}\;\mathrm{HB}101\;\mathrm{transgenics}\\ f=\mathrm{fraction}\;\mathrm{transgenic}\;\mathrm{on}\;\mathrm{DA}837\\ N_{D},N_{H}=\mathrm{total}\;\mathrm{worms}\;\mathrm{counted}\;\mathrm{on}\;\mathrm{DA}837\;\mathrm{and}\;\mathrm{HB}101\;\mathrm{plates}\\ \mathrm{SE}M_{r}=\mathrm{approximate}\;\mathrm{standard}\;\mathrm{error}\;\mathrm{of}\;\mathrm{the}\;\mathrm{mean}\;\mathrm{of}\;r \end{array}$$

The absolute value of *r* cannot be directly interpreted, but it can be compared from experiment to experiment. For statistical significance, we compared the fraction of transgenics on HB101 and DA837 with the χ^2^ test of independence.

### Integration of extrachromosomal arrays

The protocol for integrating extrachromosomal arrays (for Figure 3B) was adapted from a protocol previously described (Mello and Fire 1995). Approximately 100 transgenic L4 hermaphrodites were irradiated with 6500 rads of γ radiation from a ^137^Cs source. After approximately five to six generations, 50−100 GFP-carrying transgenic worms were individually plated to isolate integration lines that produce 100% transgenic progeny.

## Results

### Isolation of suppressors of *eat-5*

**Figure 1:**
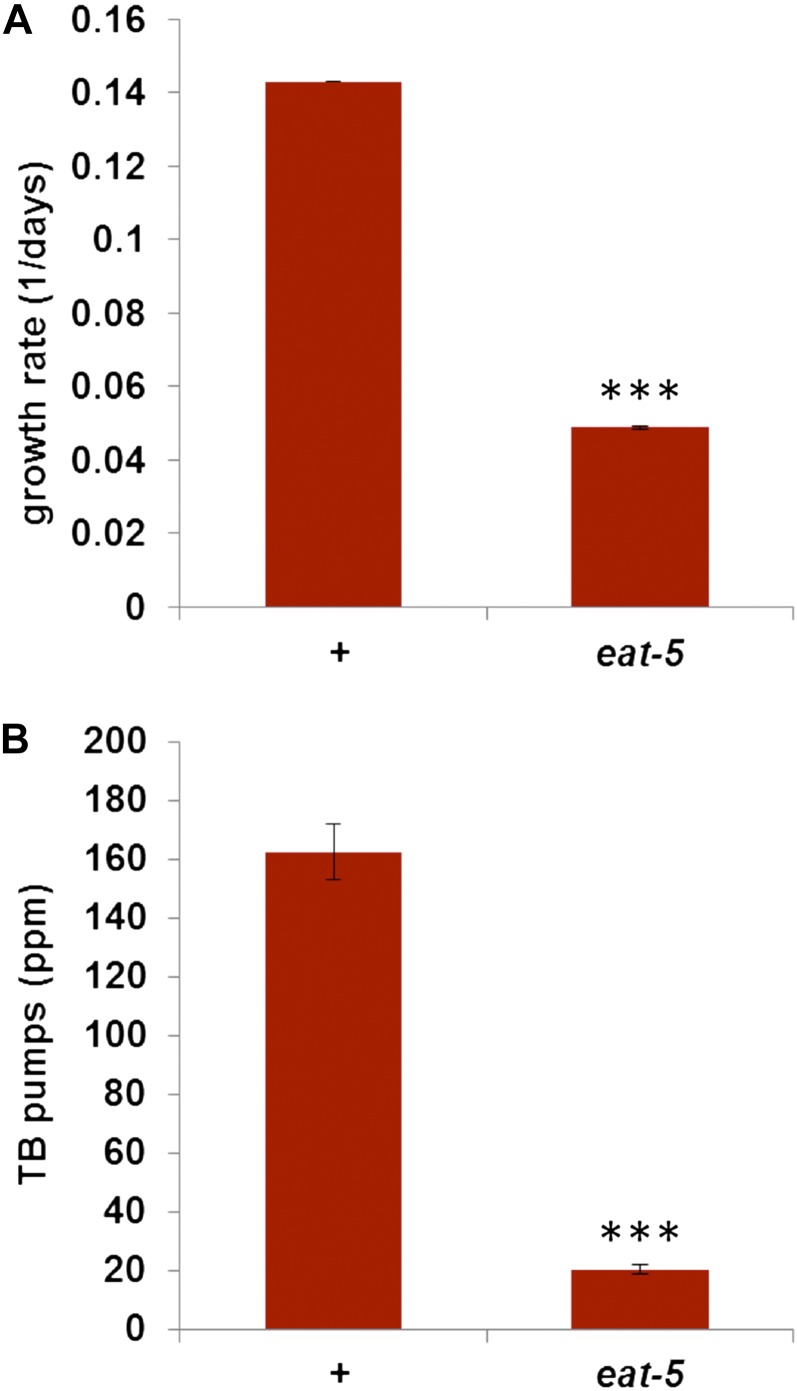
*eat-5* mutants grow and contract the TB more slowly on DA837. (A) *eat-5* grows significantly more slowly on DA837 than wild type. Growth rate is defined as the inverse of the time required for worms to consume all food. *n* = 5 for wild type, 4 for *eat-5*. (B) *eat-5* L1s pump the TB more slowly on DA837 than wild type. *n* = 6 for wild type, 12 for *eat-5*. ***Significantly different from wild type, *P* < 0.001, Student *t* test.

On *E. coli* HB101 *eat-5* null mutants grow almost as well as the wild type, although some adults have a small, pale, starved appearance. On *E. coli* DA837, however, *eat-5* worms grow very poorly. The time required to eat all the bacteria on a standard plate is about three times that of the wild type: normal worms exhaust the food in a week, whereas for *eat-5* mutants, it may take 3 wk (Figure 1A). On DA837 newly hatched *eat-5* arrests development at the first larva stage (L1), presumably because they are unable to take in any food. A few L1s eventually escape arrest, but the time varies. On a typical plate started with a single hermaphrodite, a few progeny that have escaped may be seen after a few days. This finding contrasts with wild-type worms on DA837 or either genotype on HB101, where after the same length of time hundreds of growing progeny can be seen, as well as a rapidly increasing second generation. (It may seem surprising that such a profound block of development decreases growth by only a factor of three, but growth rate is proportional to the logarithm of the brood size. A factor of three decrease in growth rate is consistent with a decrease in effective brood size from 300 to 7.) Once they escape arrest, *eat-5* worms grow almost as well as wild type. This may be because of the rapid growth of the pharynx during the L1 stage—fluid dynamic modeling suggests that the pharynx most efficiently transports particles whose diameter is substantially smaller than that of the pharyngeal lumen (Avery and Shtonda 2003). We don’t completely understand why *eat-5* worms have such a hard time with DA837. DA837 is slightly worse than HB101 for most *C. elegans* strains, but only *eat-5* mutants show a near-total L1 arrest (Avery and Shtonda 2003).

**Figure 2:**
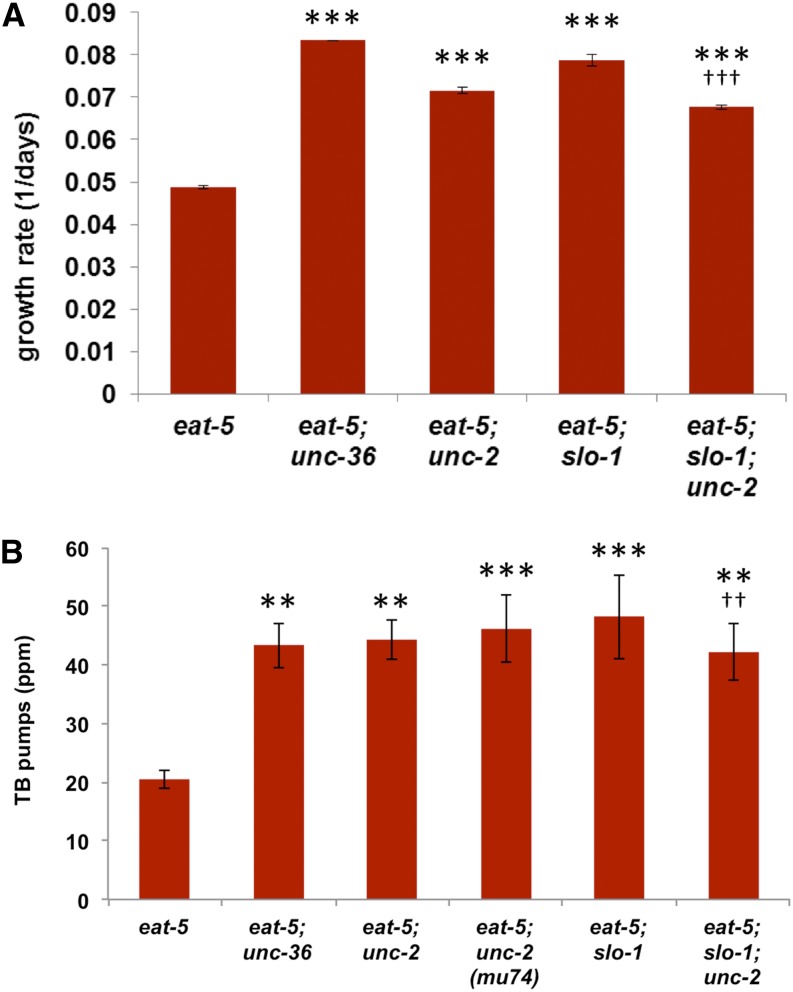
Mutations in *unc-2*, *unc-36*, and *slo-1* rescue *eat-5*. (A) *unc-36*, *unc-2*, and *slo-1* improve *eat-5* growth on DA837. Growth rate is measured as the inverse of the time required for worms to consume all food. *n* = 4 for *eat-5*, *eat-5; unc-36*, *eat-5; slo-1*, 5 for *eat-5; unc-2* and *eat-5; slo-1; unc-2*. (B) *unc-36*, *unc-2*, and *slo-1* increase L1 terminal bulb pump rate in the *eat-5* background. For both growth and pumping, the effect of *unc-2* and *slo-1* together is no greater than that of either mutation alone. *n* = 12 for all strains. **Significantly different from *eat-5*, *P* < 0.01, analysis of variance (ANOVA) + Dunnett post-tests. ***Significantly different from *eat-5*, *P* < 0.001, ANOVA + Dunnett post-tests. ^††^Significant interaction between *unc-2* and *slo-1*, *P* < 0.01, two-way ANOVA. ^†††^Significant interaction between *unc-2* and *slo-1*, *P* < 0.001, two-way ANOVA.

Because *eat-5* growth arrest occurs at the L1 stage, we compared pharyngeal pumping in *eat-5* and wild-type L1s. As previously reported, contractions of the anterior pharynx (the corpus) and posterior pharynx (the TB) are tightly synchronized in wild-type but not *eat-5* (Avery 1993; Chiang *et al.* 2006). Most corpus contractions in *eat-5* are not accompanied by TB contractions; consequently, the TB pumps more slowly than the corpus and much more slowly than the wild-type TB (Figure 1B, File S1, and File S2). We showed previously that mutations of the BK calcium-activated potassium channel *slo-1* gene increase TB pumping in *eat-5* mutants and allow better growth on DA837 (Chiang *et al.* 2006). *slo-1* does not restore synchrony between corpus and TB. Rather, it activates the M4→TB neuromuscular synapse (Chiang *et al.* 2006), providing an independent source of excitation for the TB muscle and approximately doubling TB pump rate (Figure 2B and File S3). *eat-5; slo-1* L1s escape arrest on DA837 more frequently than *eat-5* and consequently grow better, although not at wild-type rates (Figure 2A).

SLO-1 is known to inhibit synaptic transmission at body muscle neuromuscular junctions (Wang *et al.* 2001). We therefore predicted that suppressors of *eat-5* might include other genes whose products inhibit synaptic transmission. In a selection covering approximately 17,000 EMS-mutagenized haploid genomes (Table S1 and Figure S1) and a screen of 10,256 genomes, we found and analyzed 38 suppressors identifying about a dozen complementation groups (Table S2). We have identified seven of these. As expected, we found *slo-1* mutations. Two very weak suppressors, *eat-2* and *eat-18*, are known to be important for normal rates of corpus pumping (McKay *et al.* 2004). These mutations slow down corpus pumping—their isolation suggests that *eat-5* arrest on DA837 may owe something to the mismatch between corpus and TB pumping rates and not be entirely a function of slow TB pumping *per se*. One, *cfi-1*, largely restores corpus and TB synchrony and encodes a transcriptional repressor expressed in pharyngeal muscle (Shaham and Bargmann 2002). We speculate that this mutation may allow the expression of an innexin that can substitute for EAT-5 in coupling the corpus to the TB. These are all loss-of-function mutations. We also identified a likely gain-of-function mutation in the gene *dod-6*, whose expression is induced by starvation (Uno *et al.* 2013). Because starvation causes increased pumping (Avery and Horvitz 1990), this suggests that the mutation might inappropriately activate a starvation-dependent mechanism for exciting the TB. The two remaining genes, *unc-2* and *unc-36*, are the subject of the rest of this paper.

### *unc-2* and *unc-36* interact genetically with *slo-1* to suppress *eat-5*

*unc-2* and *unc-36* encode the α_1_ and α_2_/δ polypeptides respectively of the *C. elegans* homolog of vertebrate N, P/Q, and R-type voltage-gated calcium channels (Schafer and Kenyon 1995; Bargmann 1998; Mathews *et al.* 2003). We isolated two new alleles of each gene in our screen. In addition, double mutants of *eat-5* with existing *unc-2* alleles *e55* or *mu74* or *unc-36* allele *e251* showed similar suppression (Figure 2). This result was unexpected, because UNC-2/UNC-36 is generally thought of as the source of synaptic Ca^2+^ to stimulate synaptic vesicle fusion (Richmond 2005), and the phenotype of *slo-1* is opposite that of *unc-2* and *unc-36* at the body muscle neuromuscular junction (Wang *et al.* 2001; Richmond *et al.* 2001). In the *eat-5* pharynx, in contrast, all three had identical phenotypes (Figure 2). Our result suggested that at the M4→TB synapse, UNC-2/UNC-36 might inhibit synaptic transmission.

The similarity of the *unc-2* and *unc-36* phenotypes to *slo-1* suggested an explanation. There is precedent in the literature for close functional interaction and tight colocalization of N-type calcium channels and BK calcium-activated potassium channels (Roberts *et al.* 1990; Robitaille *et al.* 1993; Yazejian *et al.* 1997; Jones 1998; Marrion and Tavalin 1998; Sun *et al.* 2003). We hypothesized that in M4, Ca^2+^ that enters through UNC-2/UNC-36 activates SLO-1, thereby truncating neuronal depolarization and inhibiting vesicle fusion.

If UNC-2/UNC-36 inhibits synaptic transmission via SLO-1, *unc-2* and *slo-1* together should be no better at suppressing *eat-5* than *slo-1* alone. This prediction was confirmed (Figure 2). We also tested the effects of *slo-1* gain-of-function mutations *ky389* and *ky399* (Davies *et al.* 2003), but they were uninformative. We found to our surprise that they act like loss-of-function mutations in this context—*i.e.*, *eat-5; slo-1(ky389)* and *eat-5; slo-1(ky399)* double-mutant L1s pump faster and grow better on DA837 than *eat-5* single mutants. This result suggests that *ky389* and *ky399* may be mixomorphs [(Wright 1941a,b), cited by (Crow and Dove 1987)] that combine loss-of-function and gain-of-function effects.

### *unc-36* functions in M4 to suppress *eat-5*

We previously showed that *slo-1* is expressed in M4 (Chiang *et al.* 2006). A transcriptional fusion of the *unc-2* promoter region to GFP is expressed in a large number of neurons (Mathews *et al.* 2003) including M4 (data not shown), as well as in pharyngeal muscle (Mathews *et al.* 2003). The cellular site of action of *unc-2* and *unc-36* could therefore be any neuron or the pharyngeal muscle. To test the hypothesis that UNC-2/UNC-36 functions upstream of SLO-1 in M4 to rescue *eat-5* mutants, we targeted *unc-36* expression to M4 using the *ceh-28* (Ray *et al.* 2008) or *egl-17* (Burdine *et al.* 1998) promoter. We attempted similar experiments with *unc-2* and *egl-19* but were unable to recover worms bearing the transgenes, perhaps because the level of α_1_ subunit expression is important for the function of M4, an essential neuron (Avery and Horvitz 1987).

**Figure 3:**
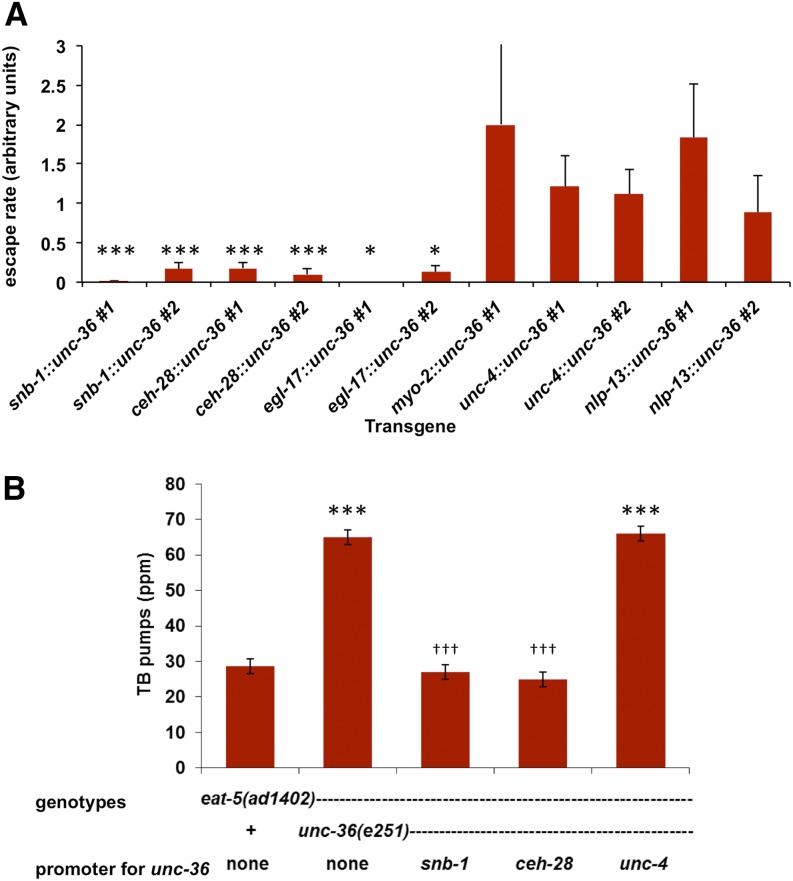
*unc-36* functions in M4 to suppress *eat-5*. (A) The ability of *eat-5; unc-36* worms carrying the transgenes shown to escape L1 arrest on DA837 was estimated as described in *Materials and Methods*. Values near 1 indicate good escape; values much less than 1 indicate rescue of the *unc-36* mutation and arrest. To clearly display the small escape rates of the rescued lines, the upper end of the error bar for *myo-2*::*unc-36 #1* has been cut off. Expression of *unc-36* under the control of a *snb-1* promoter (expressed in all neurons), a *ceh-28* promoter (expressed in M4, M2, and some extrapharyngeal cells), or an *egl-17* promoter (expressed in M4 and some extrapharyngeal cells) rescues the *unc-36* suppression of arrest. Expression of *unc-36* under the control of a *myo-2* promoter (expressed in the pharyngeal muscle), an *unc-4* promoter (expressed in I5 and extrapharyngeal cells), or an *nlp-13* promoter (expressed in M2, I1, NSM, M1, and extrapharyngeal cells) fails to rescue. Each neuronal promoter was tested in two independent transgenic lines. *Significantly different from 1, *P* < 0.05, χ^2^ test of independence with Bonferroni correction. ***Significantly different from 1, *P* < 0.001, χ^2^ test of independence with Bonferroni correction. (B) *unc-36* expression in M4 rescues *eat-5* suppression. *unc-36* increases terminal bulb pumping in the *eat-5* background. If a transgene rescues the *unc-36* suppression of *eat-5*, worms would be expected to have a terminal bulb pump rate similar to *eat-5* but different from *eat-5; unc-36*. expression of *unc-36* under the control of *snb-1* and *ceh-28* promoters fully rescued suppression. Expression of *unc-36* from an *unc-4* promoter did not rescue. TB pump rate was measured in L1s up to 4 hr after hatching. *n* = 8 for all strains. ***Significantly different from *eat-5*, *P* < 0.001, ANOVA with Tukey post-tests. ^†††^Significantly different from *eat-5*; *unc-36*, *P* < 0.001, ANOVA with Tukey post-tests.

Suppression of *eat-5* by *unc-36*, as assayed by growth on DA837 (Figure 3A) or terminal bulb pump rate (Figure 3B), was rescued by transgenic expression of wild-type *unc-36* in M4. Panneuronal expression from a *snb-1* promoter also rescued, but pharyngeal muscle expression [*myo-2* (Okkema *et al.* 1993)] or expression from promoters active in pharyngeal neurons other than M4 [*unc-4* (Miller and Niemeyer 1995) or *nlp-13* (Nathoo *et al.* 2001)] did not. This result suggests that UNC-36 is needed only in M4 to sustain the silence of the M4→TB neuromuscular junction and thus supports our hypothesis that UNC-2/UNC-36 activates SLO-1 function in M4.

### L-type but not T-type calcium channels may be needed for M4→TB transmission

There must be a source for the Ca^2+^ that stimulates vesicle fusion in M4. The fact that SLO-1, a K^+^ channel that affects synaptic transmission by making membrane potential more negative, can suppress M4→TB neuromuscular transmission, strongly suggests the involvement at this synapse, like others, of a plasma membrane voltage-gated calcium channel. But the observation that worms lacking UNC-2/UNC-36 in M4 showed increased TB pumping suggested that Ca^2+^ entry through UNC-2/UNC-36 is not necessary. Therefore, we looked for another voltage-gated calcium channel that might be doing the job. There are three voltage-gated calcium channel α_1_ genes in the *C. elegans* genome (Bargmann 1998): *unc-2* (NPQR-type), *egl-19* [L-type (Lee *et al.* 1997)], and *cca-1* [T-type (Steger *et al.* 2005)]. Two other genes with similarity to voltage-gated calcium channel α_1_ subunits, *unc-77* (also known as *nca-1*) and *nca-2*, encode a sodium leak channel (Jospin *et al.* 2007; Yeh *et al.* 2008).

**Figure 4:**
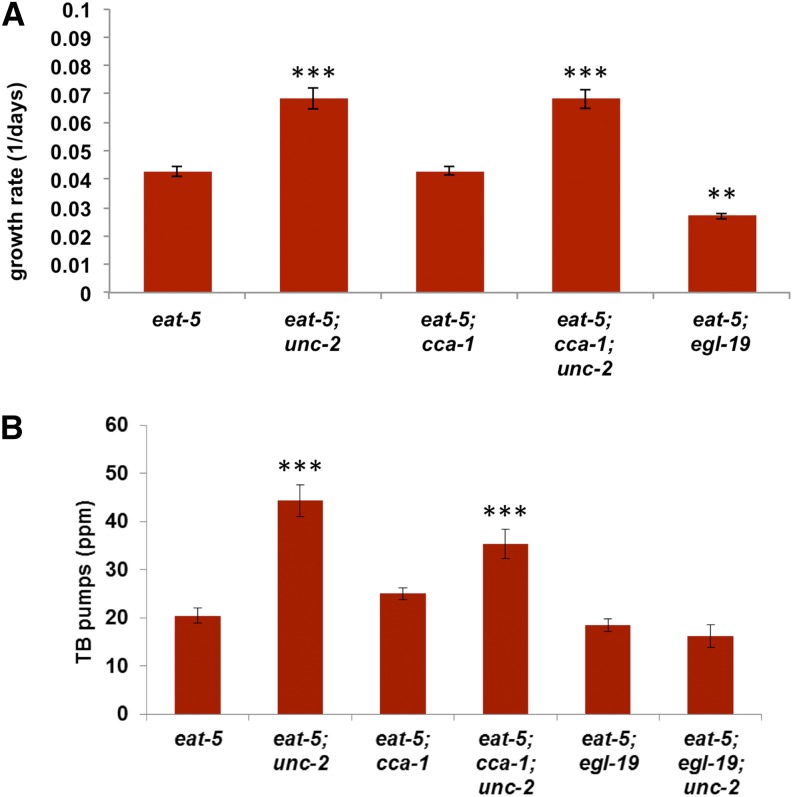
*egl-19* but not *cca-1* may be important for *unc-2* suppression of *eat-5*. (A) *unc-2* significantly increases growth rate in the *eat-5* background. *cca-1* has no effect on growth in either the *eat-5* or *eat-5; unc-2* background. A partial loss-of-function mutation in *egl-19* slightly reduces the growth rate of *eat-5*. *n* = 4 for *eat-5*, and *eat-5; egl-19*, 5 for *eat-5; unc-2*, *eat-5; cca-1*, and *eat-5; unc-2 cca-1*. (B) *unc-2* significantly increases terminal bulb pump rate in the *eat-5* background. *cca-1* has no effect on terminal bulb pump rate in the *eat-5* background and is not necessary for the *unc-2* suppression of *eat-5*. In contrast, an *egl-19* partial loss-of-function mutation reverses the *unc-2* suppression of *eat-5* while having no effect in an *unc-2(+)* background. *n* = 12 for *eat-5*, *eat-5; unc-2*, *eat-5; unc-2 cca-1*, and *eat-5; egl-19; unc-2*, 9 for *eat-5; cca-1*, and 10 for *eat-5; egl-19*. **Significantly different from *eat-5*, *P* < 0.01, ANOVA with Dunnett post-tests. ***Significantly different from *eat-5*, *P* < 0.001, ANOVA with Dunnett post-tests.

The T-type voltage-gated calcium channel gene *cca-1* is expressed in the motor neuron M4, some other pharyngeal neurons and the pharyngeal muscle (Steger *et al.* 2005). It plays a role in the response of the pharyngeal muscle to neuronal stimulation by motor neuron MC (Steger *et al.* 2005). However, a *cca-1* null mutation had no effect on either growth rate or L1 TB pumping in either the *eat-5* or the *eat-5; unc-2* background (Figure 4). In particular, *eat-5; unc-2cca-1* mutants had DA837 growth and L1 TB pumping rates not significantly different from *eat-5; unc-2* (Figure 4; *P* > 0.05) and greater than those of *eat-5* (*P* < 0.001).

This obviously implicated the L-type channel EGL-19, because it was the only voltage-gated calcium channel present in the triple mutant worms. In fact, a partial loss-of-function mutation in *egl-19* (Lee *et al.* 1997) completely blocked the *unc-2* increase in TB pumping rate (Figure 4B). *eat-5; egl-19* pumped the TB at the same rate as *eat-5*, consistent with our previous observation that the M4→TB synapse is silent in normal worms (Chiang *et al.* 2006). *eat-5; egl-19; unc-2* pumped at the same rate as *eat-5* and *eat-5; egl-19*, which is consistent with the hypothesis that *egl-19* effect on *eat-5* TB pump rate was entirely due to effects at the M4→TB synapse.

These observations are all the more striking because even a partial loss of *egl-19* function was sufficient to block the effect of *unc-2*. We were unable to test *egl-19* null mutations, because it is an essential gene, necessary for muscle contraction (Lee *et al.* 1997). *eat-5; egl-19(gf)* doubles proved similarly uninformative, as they were too unhealthy to work with, presumably because of the combined effects of *eat-5* and *egl-19(gf)* mutations on TB motions (Lee *et al.* 1997). *eat-5; egl-19* worms grew more slowly than *eat-5*. This, unfortunately, is an uninformative result, since *egl-19* mutant worms do not lay eggs (all eggs hatch internally) and therefore produce fewer progeny than wild-type. Similarly, the strong interaction between *unc-2* and *egl-19* (Schafer *et al.* 1996) made it impractical to measure the growth rate of the *eat-5; egl-19; unc-2* triple mutant.

## Discussion

### *eat-5* suppressors

We isolated mutations that suppress the slow growth phenotype of *eat-5* on DA837. These mutations defined about a dozen complementation groups (Table S2). Several of these groups are defined by only one allele, so it is likely there are more to be found. We have identified seven suppressor genes. Although we don’t have a complete description of the mechanism in every case, it is clear that they act in diverse ways.

Three of the genes, *unc-2*, *unc-36*, and *slo-1*, appear to act in a common pathway, as evidenced, for instance, by the fact that double mutants have quantitatively indistinguishable phenotypes from single mutants (Figure 2). These genes encode a BK calcium-activated potassium channel SLO-1 and an NPQR-type voltage-gated calcium channel UNC-2/UNC-36. We showed previously that SLO-1 inhibits transmission at the M4→TB muscle synapse, and we argue below that UNC-2/UNC-36 inhibits transmission by activating SLO-1. It is likely that other inhibitors of synaptic transmission can be identified by screening for mutations that activate this normally silent synapse. In fact, in a related screen (unpublished data, M. C. Cheong), we have identified mutations in *ctn-1* [α-catulin (Abraham *et al.* 2010)], *dyb-1* [dystrobrevin (Chen *et al.* 2011)], and *tom-1* [tomosyn (Gracheva *et al.* 2006, 2007)], all known inhibitors of synaptic transmission.

### Does the NPQR-type calcium channel antagonize the L-type channel?

**Figure 5:**
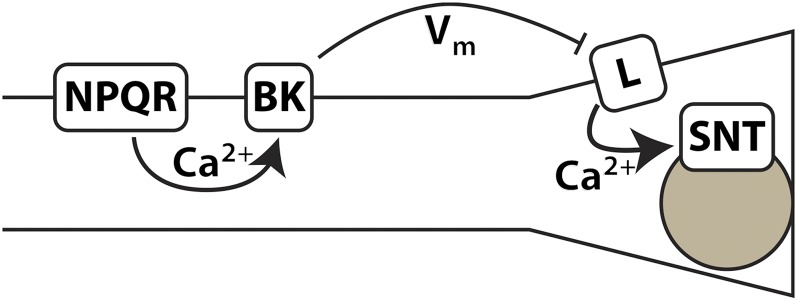
Model for BK-mediated antagonism between NPQR and L-type calcium channels in M4→TB synaptic transmission. Depolarization of M4 leads to Ca^2+^ entry through the NPQR-type voltage-gated calcium channel UNC-2/UNC-36. This Ca^2+^ activates the BK calcium-activated potassium SLO-1. The opening of the BK channel makes membrane potential become more negative, preventing or truncating the opening of L-type voltage-gated calcium channel EGL-19. Ca^2+^ entry through EGL-19 is necessary for synaptotagmin (SNT)-triggered vesicle fusion and synaptic transmission.

Figure 5 shows the simplest model that explains all our results. In this model, an initial depolarization of M4 activates the NPQR-type channel UNC-2/UNC-36, allowing Ca^2+^ entry. Ca^2+^ activates the BK channel SLO-1, which truncates the rise in membrane potential, preventing activation of the L-type voltage-gated calcium channel EGL-19. Ca^2+^ entry through the L-type channel is necessary to activate vesicle fusion at the M4→TB synapse. This model works only if there are two distinguishable Ca^2+^ signals, one that activates SLO-1, and another that activates synaptotagmin and vesicle fusion. One way to achieve this would be compartmentalization: NPQR and BK-type channels might be located in the anterior (soma and dendritic) regions of M4, with L-type channels and vesicles located in the posterior (presynaptic) region. Even in neurons as small as those of *C. elegans*, compartmentalized Ca^2+^ dynamics have been seen (Hendricks *et al.* 2012). The proposed communication between the BK channel and the L-type channel is via membrane potential rather than Ca^2+^. It is thought that *C. elegans* neurons are generally isopotential (Goodman *et al.* 1998), so membrane potential could provide the long-range signal necessary to communicate from one Ca^2+^ compartment to another. The model also requires that NPQR and L-type channels respond differently to membrane potential—this could be explained by the L-type channel opening more slowly or at a higher potential threshold than the NPQR-type.

More complicated models are also possible. For instance, it is conceivable that UNC-2/UNC-36 inhibits TB pumping in *eat-5* through an entirely distinct mechanism. Our reasons for proposing that it affects M4→TB synaptic transmission via SLO-1 are (1) We showed previously that loss of SLO-1 allows M4→TB synaptic transmission (Chiang *et al.* 2006). (2) The TB pumping and *eat-5* growth rescue phenotypes of *unc-2* and *unc-36* are indistinguishable from *slo-1* (Figure 2). (3) UNC-2 has no effect in a worm lacking SLO-1 (Figure 2). (4) *unc-36* acts in M4 to control *eat-5* growth and TB pumping (Figure 3). (5) The functional relationship between a voltage-gated calcium channel and a BK-type channel proposed is consistent with existing evidence associating neuronal voltage-gated calcium channels with BK channel function (Roberts *et al.* 1990; Robitaille *et al.* 1993; Yazejian *et al.* 1997; Jones 1998; Marrion and Tavalin 1998; Sun *et al.* 2003).

The case for an exclusive positive relationship between the L-type channel and M4→TB transmission is weaker. Our results suggest that the L-type channel is sufficient for transmission since complete elimination of the other two voltage-gated calcium channels still results in accelerated TB pumping (Figure 4). *eat-5; unc-2cca-1* worms did pump slightly slower than *eat-5; unc-2*, suggesting the T-type channel might play a minor role, but this difference was not statistically significant. The case for necessity of the L-type channel rests on the fact that *eat-5; egl-19* and *eat-5; egl-19; unc-2* pumped at the same rate as *eat-5*. This result is weak, since *egl-19* is also expressed in and necessary for pharyngeal muscle contraction. We used a hypomorphic allele of *egl-19* that had no effect on the frequency of pharyngeal muscle contraction, but we cannot exclude the possibility that its block of the positive effect of *unc-2* depended on its activity in muscle. The best experiment to test this would be expression of *egl-19* under the control of M4 and muscle-specific promoters to determine whether M4 expression is sufficient to rescue.

## Supplementary Material

Supporting Information
