# Supplementary material for: Regulation of Synaptic Transmission at the Caenorhabditis elegans M4 Neuromuscular Junction by an Antagonistic Relationship Between Two Calcium Channels
Source: G3 (Bethesda). 2014 Nov 4;4(12):2535–43. doi: 10.1534/g3.114.014308 (PMC4267947; doi:10.1534/g3.114.014308)
Supplement: Supporting Information [file supp_4_12_2535__index.html]

Regulation of Synaptic Transmission at the Caenorhabditis elegans M4 Neuromuscular Junction by an Antagonistic Relationship Between Two Calcium Channels — Supporting Information 

# Regulation of Synaptic Transmission at the *Caenorhabditis elegans* M4 Neuromuscular Junction by an Antagonistic Relationship Between Two Calcium Channels

## Supporting Information for Steciuk *et al.*, 2014

**Files in this Data Supplement:**

- Supporting Information - Figure S1, Tables S1-S2, and Files S1-S3 (PDF, 829 KB)
- Figure S1 - Time to starvation in selections. (PDF, 392 KB)
- Table S1 - Statistics for *eat-5* suppressor selection. (PDF, 215 KB)
- Table S2 - Complementation groups. (PDF, 138 KB)
- File S1 - Wild-type L1 pharyngeal pumping. (.m4v, 13 MB)
- File S2 - *eat-5* L1 pharyngeal pumping. (.m4v, 13 MB)
- File S3 - *eat-5; slo-1(ad1614)* pharyngeal pumping. (.m4v, 12 MB)
