# Supplementary material for: Regulation of Synaptic Transmission at the Caenorhabditis elegans M4 Neuromuscular Junction by an Antagonistic Relationship Between Two Calcium Channels
Source: G3 (Bethesda). 2014 Nov 4;4(12):2535–43. doi: 10.1534/g3.114.014308 (PMC4267947; doi:10.1534/g3.114.014308)
Supplement: Supporting Information [file supp_g3.114.014308_014308SI.pdf]

G3 · Genes | Genomes | Genetics

Regulation of synaptic transmission at the *C. elegans* M4 neuromuscular junction by an antagonistic relationship between two calcium channels

Steciuk, Mark*[1], Cheong, Mi Cheong[§], Waite, Christopher[§], You, Young-Jai[†], Avery, Leon[§]

*Department of Molecular Biology, University of Texas Southwestern Medical Center, Dallas, TX 75390-9148
[§]Department of Physiology and Biophysics, Virginia Commonwealth University, Richmond, Virginia, 23298-0551
[†]Department of Biochemistry and Molecular Biology, Virginia Commonwealth University, Richmond, Virginia, 23298-0614
[1]Current address: 800 Prudential Drive, Pathology Department, Baptist Health, Jacksonville, FL 32207

CORRESPONDING AUTHOR:

Leon Avery
Department of Physiology and Biophysics
MMRB 2044
1220 E Broad St
Richmond, VA 23298-0551
lavery3@vcu.edu
(804) 628-2296

DOI: 10.1534/g3.114.014308

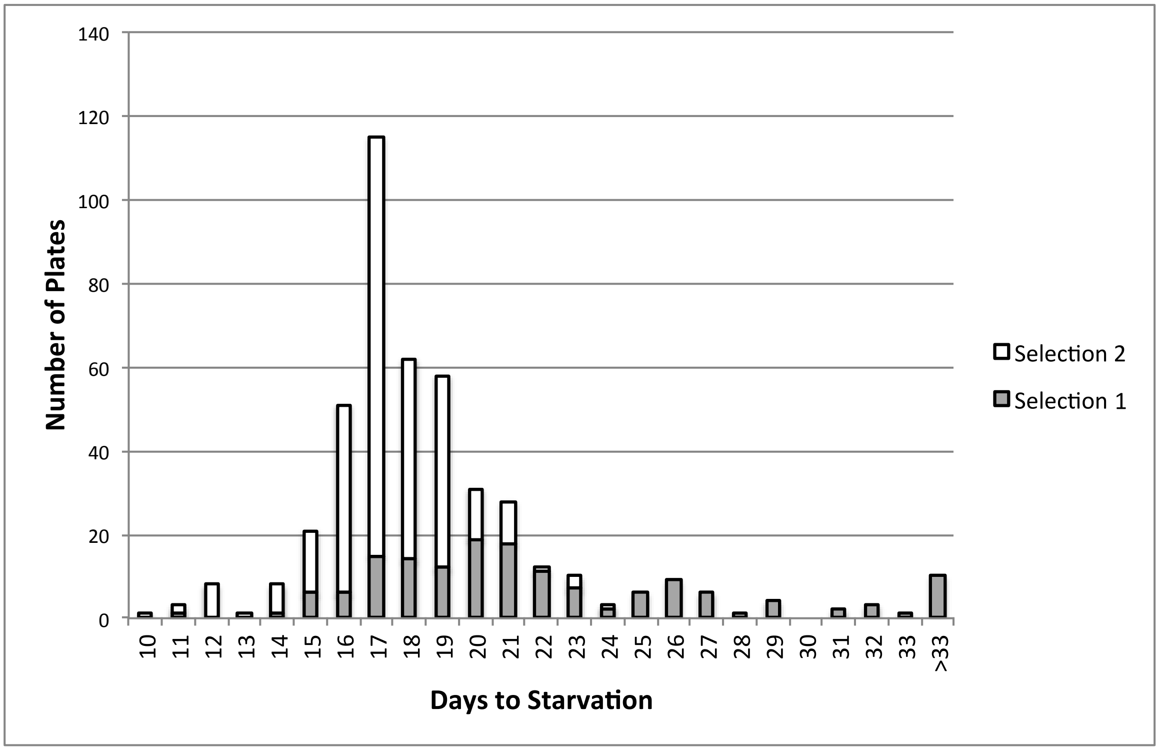

Figure S1 Time to starvation in selections. Histogram of time to starvation for the two strong suppressor selections. F2 eggs were isolated after mutagenesis of eat-5 parents and 125 (49 viable) were placed on 160 plates (Selection 1) or 240 (130 viable) on 300 plates (Selection 2). We then monitored the plates until the food was exhausted or 33 days had passed. The small peak from 10 – 12 days arises from plates that received an egg with a suppressor mutation.

Table S1 Statistics for *eat-5* suppressor selection

				recessive				dominant			
	P0	F1	F2	*μ*	*P*	*N*	*n*	*μ*	*P*	*N*	*n*
1	180	18,000	7,900	0.11	0.10	3,700	1.06	0.33	0.28	10,000	1.17
2	1,500	11,500	39,000	0.85	0.57	13,000	1.48	2.54	0.92	21,000	2.76
**Σ**	**1,700**	**30,000**	**47,000**			**17,000**				**31,000**	

μ is the mean number of phenotypically mutant F2s per mutation in the F1, $\frac{\mathrm{F2}}{4\mathrm{F1}}$ (recessive) or $\frac{3\mathrm{F2}}{4\mathrm{F1}}$ (dominant). *P*, the probability that a mutant present in the F1 is detected, is $1- e^{-m}$. *N*, the effective number of genomes screened, is $2\mathrm{F1}´\ P$. *n*, the mean number of times a mutation is isolated if it is found at all, is $\frac{m}{P}$.

Table S2 Complementation groups

group	gene	mutations*			strength	other phenotypes	inheritance†	linkage
1	*cfi-1*	13	9	4	strong		AR	I
	dod-6	3	3	0	strong	molting defects	AD	III
2	*slo-1*	6	1	5	weak	loopy movement	AR	V
3	*unc-2*	2	0	2	weak	Unc	XR	X
4	*unc-36*	2	0	2	weak	Unc	AR	III
5	*eat-2*	1	0	1	weak	slow pumping	AR	II
6	unknown	2	0	2	weak		AR	
7	*eat-18*	1	0	1	weak	Unc‡	AR	I
8	unknown	3	0	3	weak		AR	
9	unknown	1	0	1	weak		AR	
10	unknown	1	0	1	weak		AR	
11	unknown	1	0	1	weak		AR	
12	unknown	1	0	1	weak		AR	

*Columns are the total number of mutations isolated, the number isolated in the DA837 growth selection, and the number isolated in the L1 arrest escape screen.

†AD = autosomal dominant, AR = autosomal recessive, XR = X-linked recessive.

‡Since other existing *eat-18* mutations suppress *eat-5* but do not have an Unc phenotype, it is likely that this phenotype is caused by a second mutation irrelevant to the Sef phenotype.

Files S1-S3

Supplemental videos

Available for download at http://www.g3journal.org/lookup/suppl/doi:10.1534/g3.114.014308/-/DC1

File S1 Wild-type L1 pharyngeal pumping

File S2 *eat-5* L1 pharyngeal pumping

File S3 *eat-5; slo-1(ad1614)* pharyngeal pumping
