# Supplementary material for: Regulation of Synaptic Transmission at the Caenorhabditis elegans M4 Neuromuscular Junction by an Antagonistic Relationship Between Two Calcium Channels
Source: G3 (Bethesda). 2014 Nov 4;4(12):2535–43. doi: 10.1534/g3.114.014308 (PMC4267947; doi:10.1534/g3.114.014308)
Supplement: Supporting Information [file supp_g3.114.014308_FigureS1.pdf]

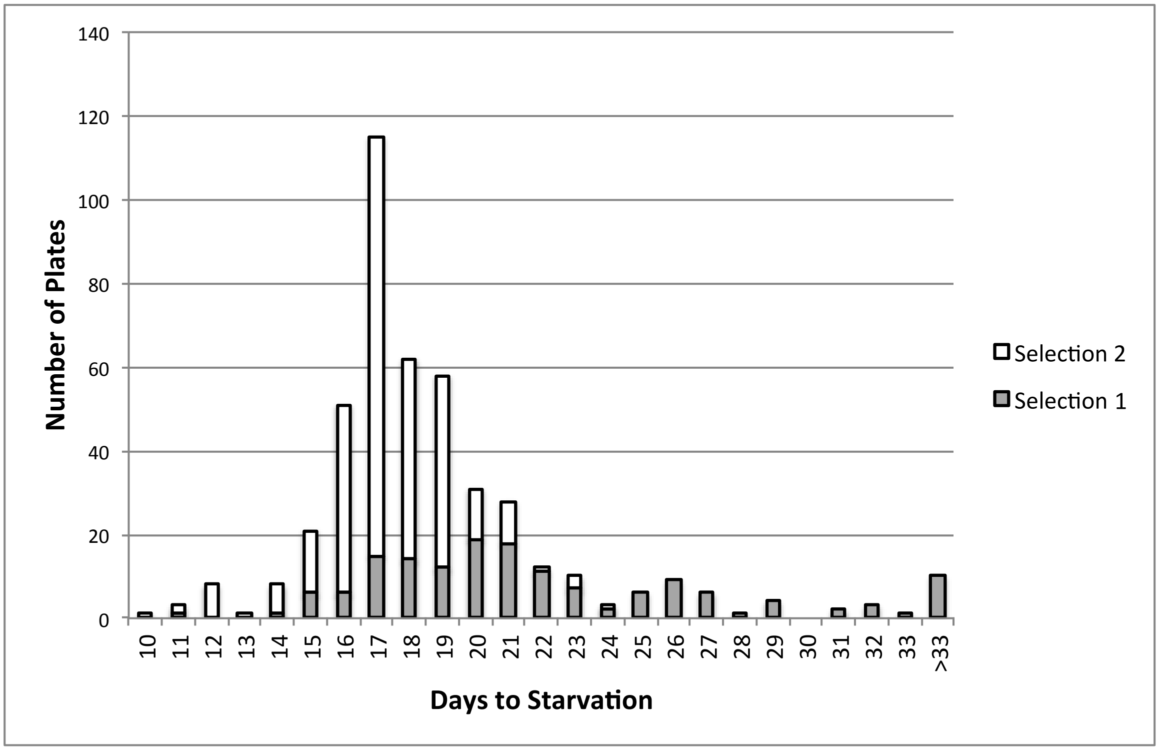

Figure S1 Time to starvation in selections. Histogram of time to starvation for the two strong suppressor selections. F2 eggs were isolated after mutagenesis of eat-5 parents and 125 (49 viable) were placed on 160 plates (Selection 1) or 240 (130 viable) on 300 plates (Selection 2). We then monitored the plates until the food was exhausted or 33 days had passed. The small peak from 10 – 12 days arises from plates that received an egg with a suppressor mutation.
