# Supplementary material for: Regulation of Synaptic Transmission at the Caenorhabditis elegans M4 Neuromuscular Junction by an Antagonistic Relationship Between Two Calcium Channels
Source: G3 (Bethesda). 2014 Nov 4;4(12):2535–43. doi: 10.1534/g3.114.014308 (PMC4267947; doi:10.1534/g3.114.014308)
Supplement: Supporting Information [file supp_g3.114.014308_TableS1.pdf]

Table S1 Statistics for *eat-5* suppressor selection

				recessive				dominant			
	P0	F1	F2	*μ*	*P*	*N*	*n*	*μ*	*P*	*N*	*n*
1	180	18,000	7,900	0.11	0.10	3,700	1.06	0.33	0.28	10,000	1.17
2	1,500	11,500	39,000	0.85	0.57	13,000	1.48	2.54	0.92	21,000	2.76
**Σ**	**1,700**	**30,000**	**47,000**			**17,000**				**31,000**	

μ is the mean number of phenotypically mutant F2s per mutation in the F1, $\frac{\mathrm{F2}}{4\mathrm{F1}}$ (recessive) or $\frac{3\mathrm{F2}}{4\mathrm{F1}}$ (dominant). *P*, the probability that a mutant present in the F1 is detected, is $1-e^{-\mu}$. *N*, the effective number of genomes screened, is $2\mathrm{F1}\times P$. *n*, the mean number of times a mutation is isolated if it is found at all, is $\frac{\mu}{P}$.
