# Supplementary material for: Regulation of Synaptic Transmission at the Caenorhabditis elegans M4 Neuromuscular Junction by an Antagonistic Relationship Between Two Calcium Channels
Source: G3 (Bethesda). 2014 Nov 4;4(12):2535–43. doi: 10.1534/g3.114.014308 (PMC4267947; doi:10.1534/g3.114.014308)
Supplement: Supporting Information [file supp_g3.114.014308_TableS2.pdf]

Table S2 Complementation groups

group	gene	mutations*			strength	other phenotypes	inheritance†	linkage
1	*cfi-1*	13	9	4	strong		AR	I
	dod-6	3	3	0	strong	molting defects	AD	III
2	*slo-1*	6	1	5	weak	loopy movement	AR	V
3	*unc-2*	2	0	2	weak	Unc	XR	X
4	*unc-36*	2	0	2	weak	Unc	AR	III
5	*eat-2*	1	0	1	weak	slow pumping	AR	II
6	unknown	2	0	2	weak		AR	
7	*eat-18*	1	0	1	weak	Unc‡	AR	I
8	unknown	3	0	3	weak		AR	
9	unknown	1	0	1	weak		AR	
10	unknown	1	0	1	weak		AR	
11	unknown	1	0	1	weak		AR	
12	unknown	1	0	1	weak		AR	

*Columns are the total number of mutations isolated, the number isolated in the DA837 growth selection, and the number isolated in the L1 arrest escape screen.

†AD = autosomal dominant, AR = autosomal recessive, XR = X-linked recessive.

‡Since other existing *eat-18* mutations suppress *eat-5* but do not have an Unc phenotype, it is likely that this phenotype is caused by a second mutation irrelevant to the Sef phenotype.
